# One-step hydrothermal synthesis of thioglycolic acid capped CdS quantum dots as fluorescence determination of cobalt ion

**DOI:** 10.1038/s41598-018-27244-0

**Published:** 2018-06-12

**Authors:** Zhezhe Wang, Xinxin Xing, Yue Yang, Rongjun Zhao, Tong Zou, Zidong Wang, Yude Wang

**Affiliations:** 1 Department of Physics, Yunnan University, 650091 Kunming, People’s Republic of China; 2 Key Lab of Quantum Information of Yunnan Province, Yunnan University, 650091 Kunming, People’s Republic of China; 3 School of Materials Science and Engineering, Yunnan University, 650091 Kunming, People’s Republic of China

## Abstract

Highly luminescent CdS quantum dots capped with thioglycolic acid (TGA@CdS QDs) were synthesized from cadmium chloride and thiourea as cadmium and sulfur sources via simple hydrothermal method. The room temperature photoluminescence (RTPL) properties of TGA@CdS QDs were investigated. The results indicate that the polarity of the solvent and the surface trap state resulted in the broadness Stokes shift between the maximum absorption wavelength and the emission wavelength of TGA@CdS QDs. The Co^2+^ sensing properties of fluorescence determination were investigated using TGA@CdS QDs. The as-synthesized CdS QDs exhibits the excellent selectivity and sensitivity of fluorescence quenching for cobalt ion (Co^2+^). The limit of detection (LOD) is as low as 0.05 μM which is much lower than maximum limit of cobalt ions in drinking water. The linear response range of Co^2+^ was from 0.5 to 80 μM. The sensing system revealed the advantages of low detection limit, excellent selectivity, high sensitivity, convenience and low cost. The color change of CdS QDs shows potential applications in the detection of Co^2+^.

## Introduction

Cobalt, as an important micronutrient, is an essential component of vitamin B_12_, and plays a vitally important role in humans and biological systems^1–3^. However, cobalt could be a toxic compound at high concentrations which could cause many diseases such as asthma, decreased cardiac output, cardiac enlargement, heart disease, lung disease, dermatitis and vasodilation^4^. With the widely applications of the cobalt salt^5–7^, large quantities of wastewater containing cobalt ions also arise. It will be caused serious harm to people, animals, plants and other microorganisms if the untreated wastewater discharged into the environment. The concentration of cobalt ions shall not exceed 1 mg/L (16.9 μM) in the wastewater^8^. The World Health Organization (WHO) recommends maximum limits of cobalt ions in drinking water of 1.7 μΜ^9^. For these reasons, it is important to detect trace amounts of Co^2+^ in the water.

So far a variety of sophisticated methods have been used to detect Co^2+^ concentrations on aqueous solution, such as atomic absorption spectrometry (AAS), high performance liquid chromatography (HPLC), inductively coupled plasma mass/emission spectroscopy, and voltammetry^10–18^. These methods are characterized by good accuracy while they in general are quite time-consuming and require expensive and complex equipment. The sample preparation and pretreatment could be cumbersome. These restrict its application range. Therefore, a rapid, simple, inexpensive and highly sensitive method for the detection of Co^2+^ is undoubtedly realistic significance.

More recently, optical sensing approaches have attracted the attention of many researchers due to their quick response, simplicity, high sensitivity and selectivity^19–25^. Luminescent semiconductor quantum dots (QDs) have high quantum yield, sharp emission spectra, broad absorption spectra, long life-times and tunable color which are superior to conventional fluorescent dyes. Recently, based on the Co^2+^–induced changes in fluorescence intensity of QDs, the various reports have been published on the using of QDs for fluorescence determination of Co^2+^^19–24^. The concentration of Co^2+^ can be measured through the change of fluorescence intensity of QDs caused by the interactions between QDs surface and Co^2+^. Besides, Some papers have reported that heavy metal ion, such as Hg^2+^, Ag^+^, Cu^2+^, Co^2+^and Ni^2+^, have been detected by surface-modified CdS QDs based on the gradual quench of the fluorescence intensity of the QDs^25–28^. Previous reports have demonstrated monitoring the optical changes of QDs fluorescence by Co^2+^ may provide a simple method to develop QD-based fluorescence probes. However, these studies revealed weak abilities of anti-interference and high detection limit. So, the sensitivity of selectivity of the related sensor based fluorescence QDs still had the large space to improve by the stability of CdS QDs. TGA could improve the performance of CdS QDs in aqueous solutions because of the passivation and hydrophilicity properties. Hence, TGA as the surface modification would enhance the stability of CdS QDs as well as the selectivity for metal ions in aqueous solutions.

In this work, the low-cost TGA-capped CdS QDs (TGA@CdS QDs) was prepared from cadmium chloride and thiourea as cadmium and sulfur sources by hydrothermal method without removing oxygen during the preparation process. The obtained TGA@CdS QDs exhibited excellent selectivity and sensitivity for Co^2+^ by fluorescence quenching. The fluorescence quenching mechanism and the fluorescence generation mechanism of the TGA@CdS QDs was also studied.

## Experimental

### Reagents and materials

The materials used in the experiments included cadmium chloride hydrate (CdCl_2_·2.5H_2_O 99%), thioglycolic acid (TGA, 90.0%), thiourea (99%), sodium hydroxide (NaOH, 99%) and various metal salts. All the chemical reagents used in the experiments were obtained from commercial sources as guaranteed-grade reagents and used without further purification. All solutions were prepared with deionized water. The concentration of the QDs was 0.012 M calculated based on the original cadmium source.

### Characterization

Photoluminescence spectra were measured at room temperature with an imaging spectrometer (iHR320 HORIBA Jobin Yvon Inc., Edison, NJ, USA.). The slit width used for both excitation and emission was 2 nm. Transmission electron microscopy (TEM), high resolution transmission electron microscopy (HR-TEM), and selected area electron diffraction (SAED) with energy dispersive X-ray (EDX) measurements were performed by JEOL JEM-2100 with an acceleration voltage of 200 kV. The microstructure of the synthesis CdS QDs was analyzed by TEM and HR-TEM and the crystal structure was obtained by SAED. The surface topography was investigated using a SPA-400 SPM atomic force microscope (AFM). The UV-vis absorption spectra were obtained at an UV-1800 spectrophotometer of Jinghua Instruments with a wavelength range between 200 and 800 nm. Fourier transform infrared (FTIR) spectra were recorded on AVATAR 360 FT-IR spectrophotometer in the range of 4000–5000 cm^−1^. The time resolved photoluminescence lifetime decays were measured by Hamamatsu compact fluorescence lifetime spectrometer C11367 (Quantaurus-Tau), using an LED light source with *λ*_ex_ = 365 nm. pH were measured by an inoLab pH Level 1 precision pH meter.

### Synthesis of TGA@CdS QDs

TGA@CdS QDs were prepared via a classic hydrothermal method as previously described in report^29^. Briefly, 0.7458 g cadmium chloride hydrate (CdCl_2_·2.5H_2_O) and 0.4400 g thiourea (CH_4_N_2_S) were dissolved in 280 mL deionized water and stirring for 30 min. Then 15 μL thioglycolic acid (TGA) was slowly added to the aqueous solution stirring for 30 min. The pH value of the solution was adjusted by using 1 M NaOH aqueous solution. After stirring for 1 h, the mixture solution was transferred to a 300 mL Teflon-lined stainless steel autoclave. The autoclave was maintained at 120 °C for 2 h and cooled to room temperature. The pH value of the as-synthesized solution is 7.0.

### Selective detection of Co^2+^

The fluorescence determination of Co^2+^ in aqueous solution at room temperature was achieved using the prepared TGA@CdS QDs aqueous solution based on fluorescence quenching. The QDs solution is stable and a distinct color change occurred by our eyes when the required amount of cobalt ion added to the QDs solution. The fluorescence quenching was analyzed by the maximum fluorescence intensity.

## Results and Discussion

### Characterization of the TGA@CdS QDs

**Figure 1:**
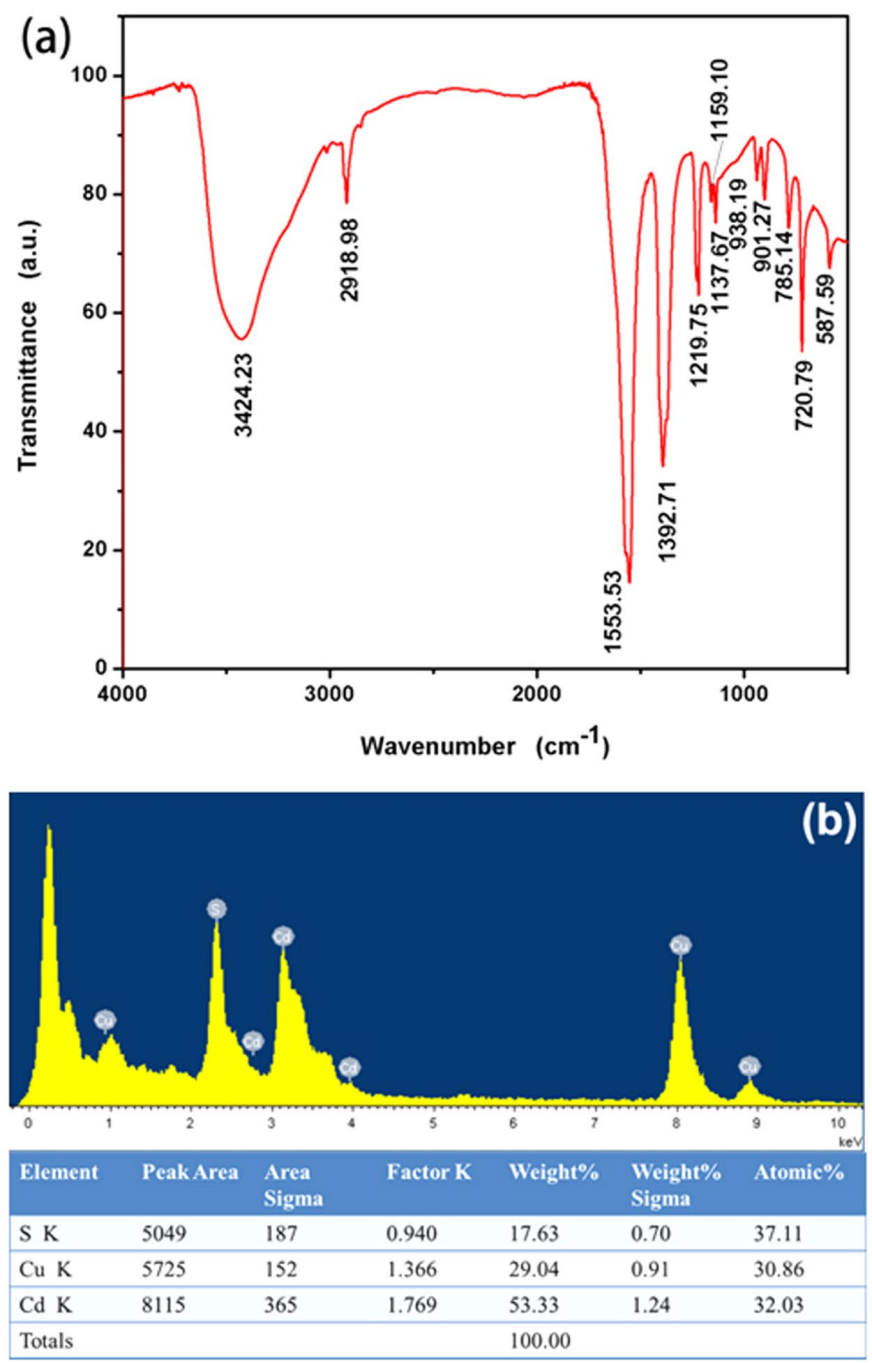
FTIR spectrum (**a**) and EDX spectrum (**b**) of the as-synthesized CdS QDs.

Figure 1(a) shows the FTIR spectrum of as-obtained TGA@CdS QDs in the range from 400 cm^−1^ to 4000 cm^−1^. The characteristic IR peaks appear at 3424, 2919, 1553, 1392, 1219, 1137 cm^−1^, respectively. The strong absorption peak is observed at 3424.23 cm^−1^ arisen from the hydroxyl bond stretching vibration of -COOH group and broaden hydroxyl bond can be ascribed to the solvent^30^. The peak at 2918.98 cm^−1^ corresponds to –CH_2_ asymmetric stretching vibration modes^31^. The strong peak at 1553.53 cm^−1^ and 1392.71 cm^−1^ correspond to asymmetric and symmetric stretching respectively in carboxylate group of TGA. The peaks at 1219.75 cm^−1^ and 1137.67 cm^−1^ are assigned to asymmetric and symmetric stretching vibrations of C-O, respectively. The absence of distinct S-H characteristic band of TGA at 2600–2550 cm^−1^ confirms the capping of CdS QDs which is due to covalent bond between cadmium of CdS and sulfur of TGA^32^. These results confirm that the synthesized QDs are well capped and stabilized with TGA molecules. The EDX spectrum (Fig. 1(b)) of the as-synthesized solution confirms the presence of CdS QDs. The atomic ratio of [Cd]:[S] is 1:1.16. One part sulfur forms CdS QDs with cadmium, the other belongs to TGA which capped on the CdS QDs surface. The Cu peak arises from the copper nets.

**Figure 2:**
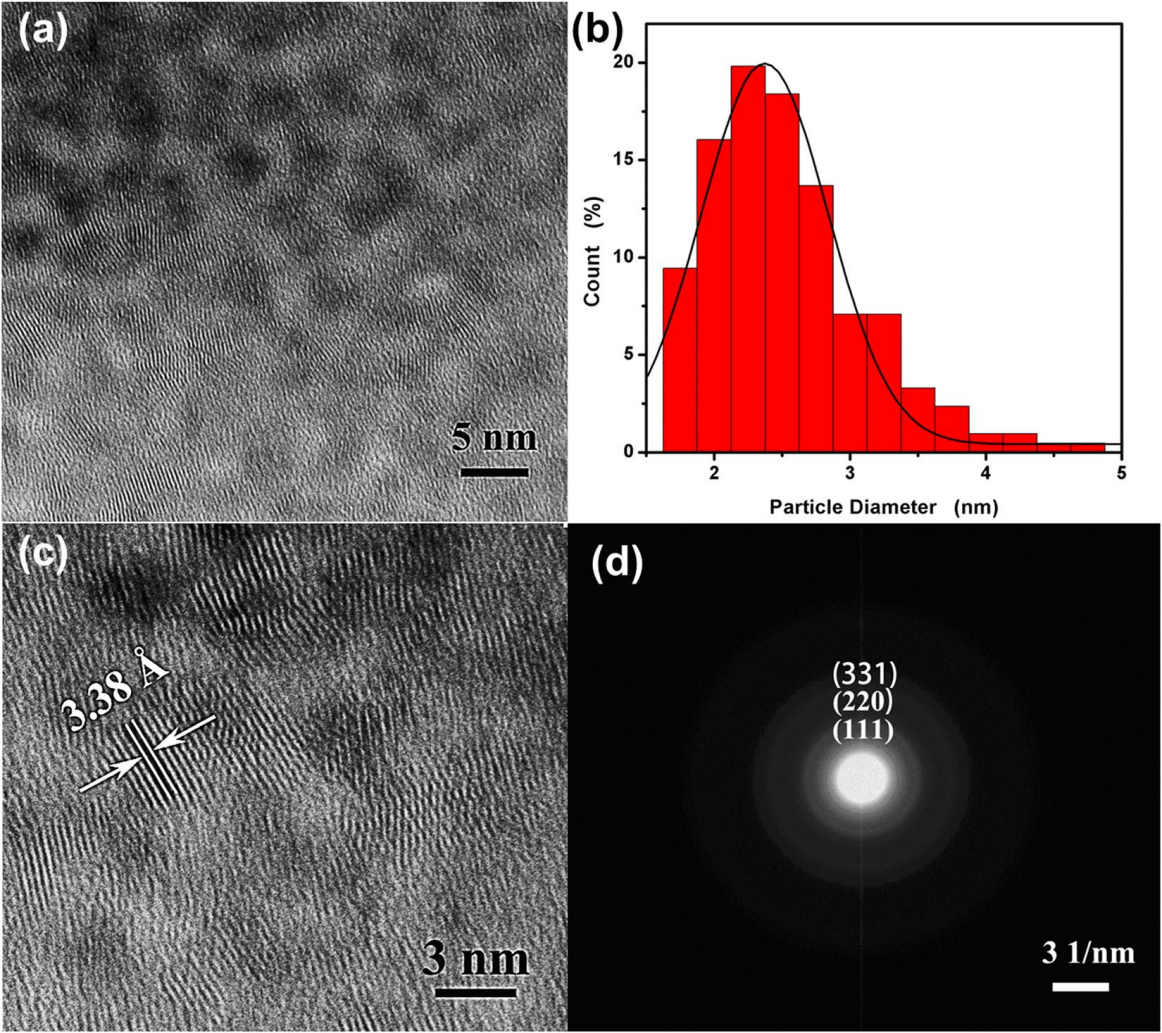
(**a**) HR-TEM image of as-synthesized CdS QDs. (**b**) statistical analysis of the sizes of as-synthesized CdS QDs. (**c**) HR-TEM images of as-synthesized CdS QDs with the lattice fringes. (**d**) The corresponding SAED pattern of the as-synthesized CdS QDs.

Figure 2 shows the HR-TEM images of the as-synthesized TGA@CdS QDs. From the HR-TEM image shown in Fig. 2(a), a number of uniformed-sized CdS QDs with distinct crystal lattice can be clearly observed. Figure 2(b) shows that the average size of stable CdS QDs is 2.37 ± 1.1 nm by measuring the diameters of more than 200 particles from the HR-TEM image. The as-synthesized quantum dots, with an interplanar spacing of 3.38 Å corresponding to the lattice plane (111) of CdS (JCPDS 10–454), as shown in Fig. 2(c). The SAED pattern (Fig. 2(d)) illustrates the presence of (111), (220), (311) planes of the cubic zinc blende structure of CdS. The EDX spectrum (Fig. 1(b)) also confirms the formation of TGA@CdS QDs with the atomic ratio of Cd to S being 1:1.16. All the analyses demonstrate that the CdS QDs was synthesized by the one-step hydrothermal method, which is simple and cost-effective.

**Figure 3:**
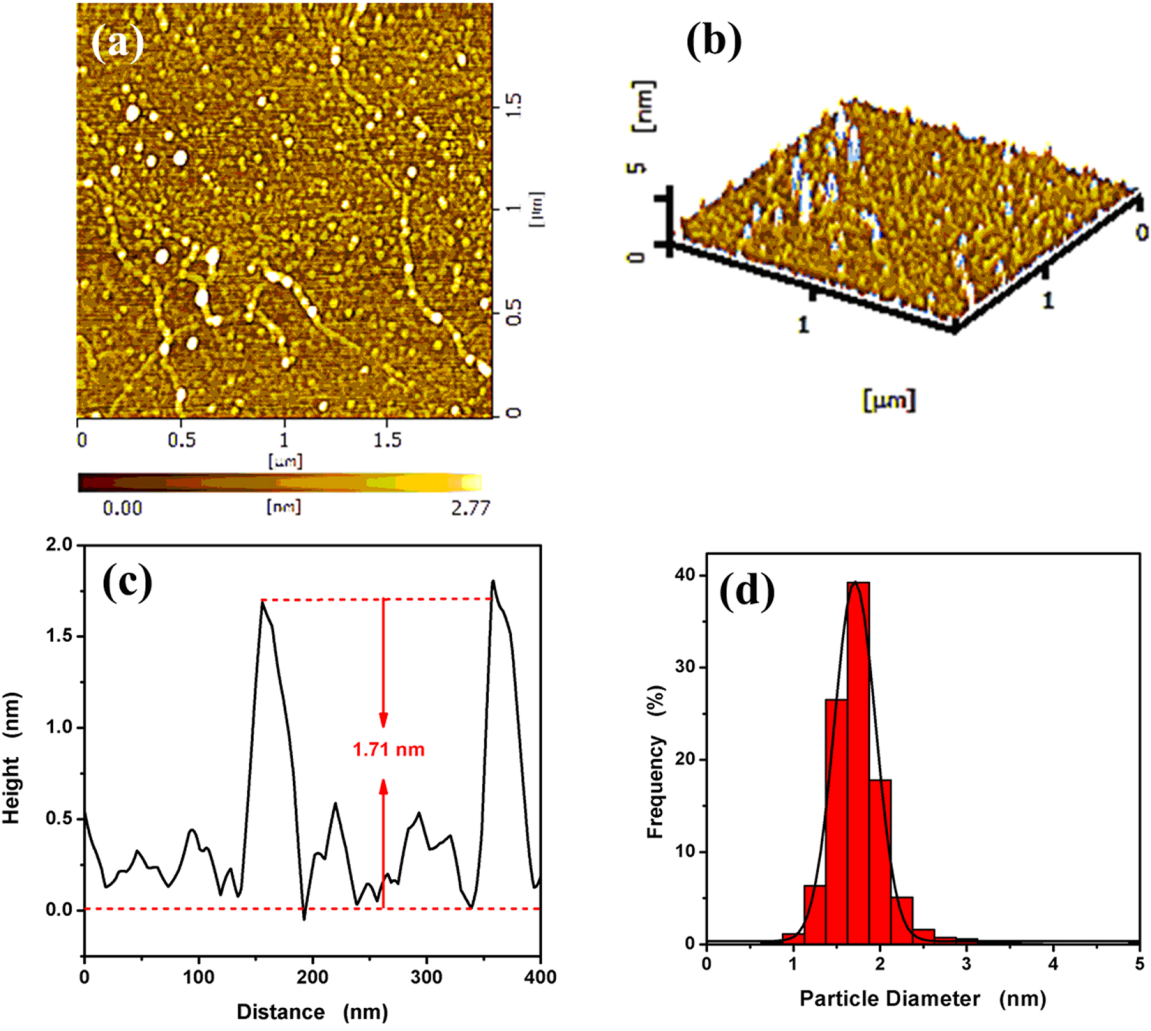
(**a**) and (**b**) represent 2D and 3D AFM images of TGA@CdS QDs deposited on silicon substrate, respectively. (**c**) Height profiles along the line in (**a**). (**d**) Statistical analysis of the heights of TGA@CdS QDs measured by AFM.

The topographic morphology of as-synthesized CdS QDs was examined further through AFM. Figure 3(a–c) show the typical two-dimensional (2D), three-dimensional (3D) AFM images, and height profile of as-synthesized CdS QDs, respectively. As shown in Fig. 3(a,b), many uniformly sized CdS QDs are observed and the height of CdS QDs is 1.71 nm approximately. The histogram of the height of the CdS QDs is exhibited in Fig. 3(d), which shows an average size of 1.71 ± 0.6 nm. In contrast of the size distribution of QDs by both TEM and AFM, it could be concluded that slightly different result. The size distribution of QDs by measured the lattice fringe image. Hence, the as-synthesized QDs is uniform and thin, which could be conductive to improve performance.

**Figure 4:**
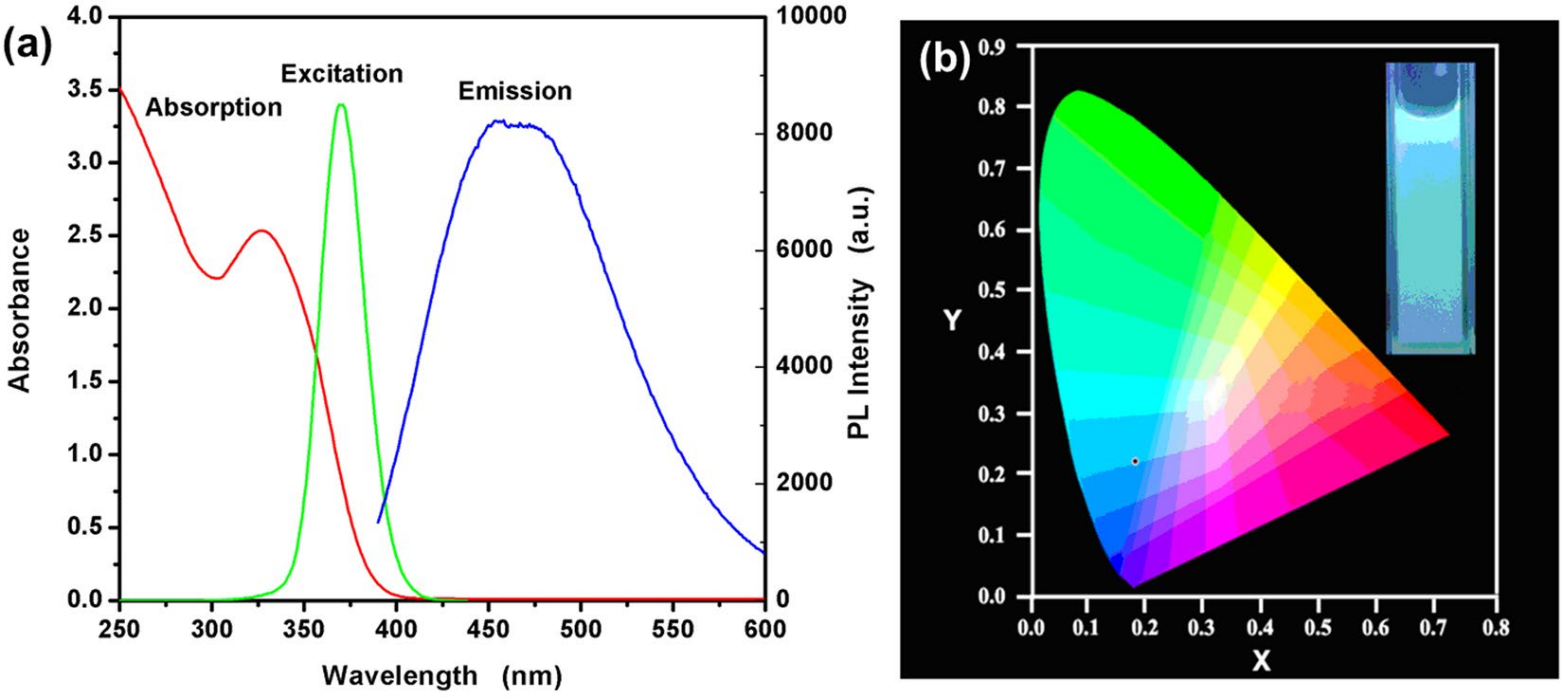
(**a**) The UV-vis absorbance (red line), photoluminescence spectra (green line) and the emission spectra (blue line) of the as-synthesized CdS QDs. (**b**) The CIE 1931 coordinates and picture of the samples under 365 nm UV lamp with a distance of 25 cm.

The absorption, emission and excitation spectra of the as-synthesized CdS QDs are show in Fig. 4(a). The broad absorption spectra can be observed in the ultraviolet range with the wavelength of 400 nm which is different from the bulk CdS^30^. This is attributed to the quantum size effects of the samples. The emission spectra indicate the maximum fluorescence intensity at 454 nm under the 370 nm excitation wavelength. Between the maximum absorption wavelength and the emission wavelength, a broadness and large Stokes shift was observed owing to the surface defect emission^25^. The characteristic is not always observed when using organic fluorophores which lead to the semiconductor quantum dots are better than the conventional organic fluorescent probes. The absorption edges (obtained by the intersection of the sharply decreasing region of the spectrum with the baseline) are located at 386 nm, which corresponds to band gap of 3.21 eV. According to the relationship between the absorption edge (*λ*_em_) and the size of QDs (Eq. (1)), the mean size of the particles can be calculated using the following equation^33^:

1 $$2{R}_{{\rm{C}}{\rm{d}}{\rm{S}}}=\frac{0.1}{(0.1338-0.0002345{\lambda }_{{\rm{e}}{\rm{m}}})}$$

Regarding Eq. (1) and absorption spectra (red line), it can be estimated that the mean diameter size of TGA@CdS QDs is 2.31 nm, corresponding to the consequence of TEM. Using the band gap value of 3.21 eV, the particles diameter was calculated following the formula of Brus (Eq. (2)) based on the effective mass approximation^34–36^.

2 $$E={E}_{{\rm{g}}}+\frac{{h}^{2}}{8{R}^{2}}(\frac{1}{{m}_{{\rm{e}}}}+\frac{1}{{m}_{{\rm{h}}}})-\frac{1.8{e}^{2}}{\varepsilon R}$$

where *E*_g_ is the band gap of the bulk material (2.42 eV), *h* is the reduced Plank’s constant (6.625 × 10^−34^ J·s), *R* is the radius of spherical nanocrystals, *e* is the charge of the electron (1.6 × 10^−19^ C), ${\varepsilon }_{0}$ is the vacuum dielectric constant (${\varepsilon }_{0}$ = 8.854 × 10^−12^ F/m), $\varepsilon$ is the semiconductor dielectric constant (5.7 ${\varepsilon }_{0}$), m_e_ and ${m}_{{\rm{h}}}$ are effective masses of the electrons and holes, respectively, and ${m}_{0}$ is the free electron mass (${m}_{0}$ = 9.108 × 10^−31^ kg). With the effective masses of electrons (${m}_{{\rm{e}}}$ = 0.19 ${m}_{0}$) and holes (${m}_{{\rm{h}}}$ = 0.8 ${m}_{0}$), the diameters of TGA@CdS QDs is found to be 1.76 nm, which agrees well with the AFM. The sample emitted greenish blue color was exhibited on the corresponding CIE coordinates (Fig. 4(b)) with a corresponding CIE coordinates are (0.18, 0.22) and the photographs of the emission under UV light (Fig. 4(b), inset) for the same samples.

**Figure 5:**
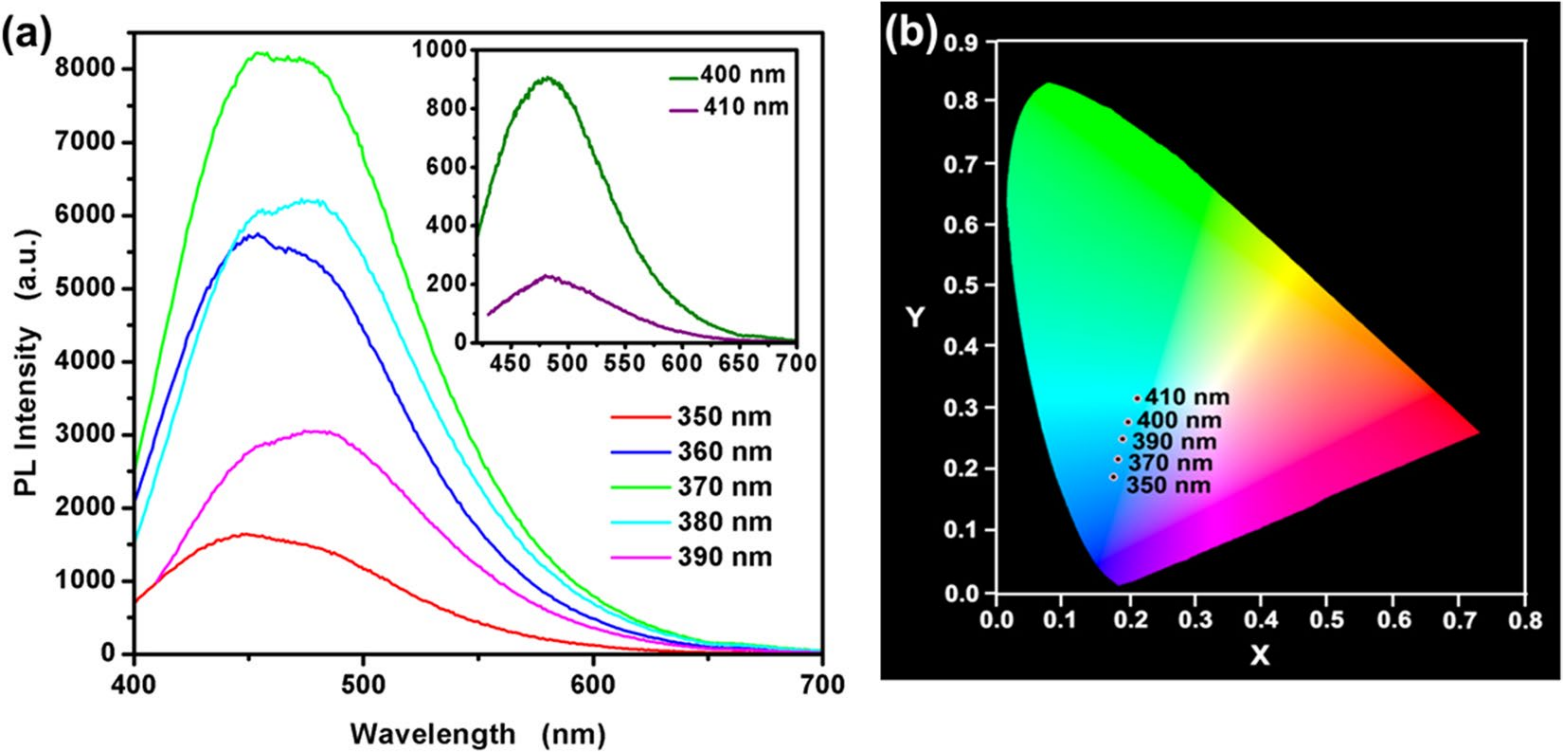
The excitation-dependent emission spectrum (**a**) and the CIE 1931 chromaticity chart (**b**) of TGA@CdS QDs.

Figure 5(a) shows that the emission peak almost unchanged with the variation of excitation wavelength in the range of 350–390 nm. The photoluminescence intensity firstly increases and then decreases with the increase of excitation wavelength. The change of fluorescence intensity can be explained that the long wave emission part of the as-synthesized CdS QDs is associated with the quantum confinement effect. The TEM and AFM images of as-synthesized CdS QDs indicate that the particle size is not identical versions. So, when the excitation wavelength gradually increases, the more CdS QDs are excited and the fluorescence intensity is also gradually enhanced after reaching the maximum. And then, the excited CdS QDs gradually reduce and the fluorescence intensity also gradually weakened. The photoluminescence spectrum shows two fluorescence peaks centered on 454 nm and 474 nm, respectively. It can be mainly viewed from two aspects: the surface defects formed in the as-synthesized CdS QDs and the size distribution of particles. There is small variation in color across the coordinates of the as-synthesized CdS QDs under the different excitation wavelength as shown in Fig. 5(b). The main reason for this is that the band-edge emission of large size CdS QDs and trap-stare emission are distinct increasingly.

**Figure 6:**
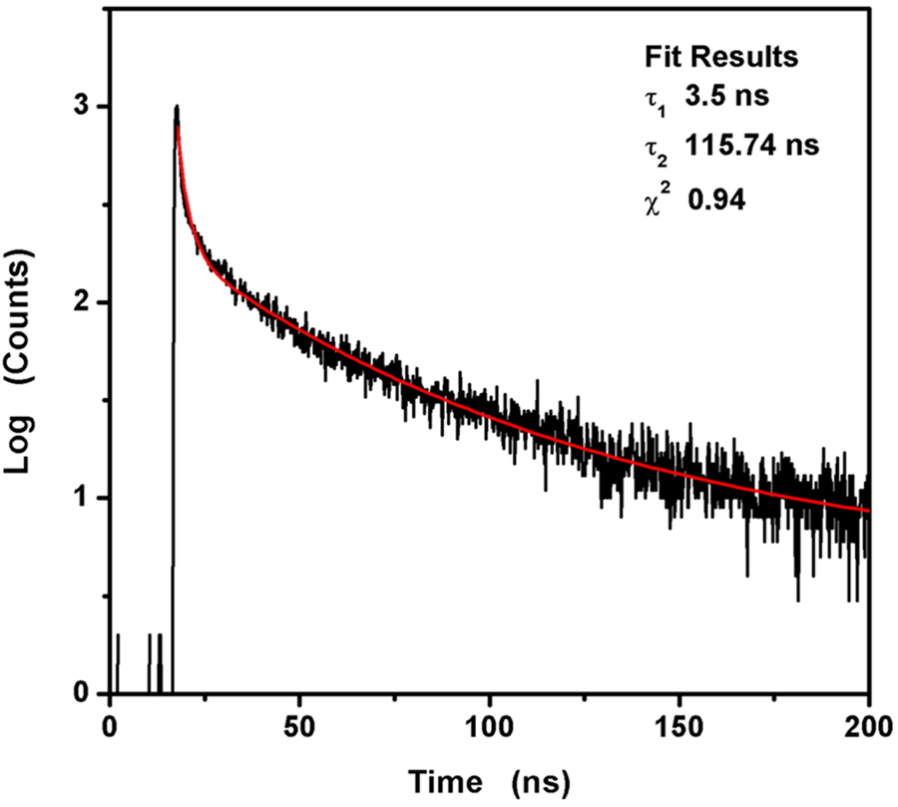
Fluorescence decay curves of as-synthesized CdS QDs (black) and the corresponding quadratic exponential fitting curve (red).

Time-resolved PL experiment (Fig. 6) shows that the lifetime of TGA@CdS QDs. The lifetime measurements were carried out immediately after preparation as well as after 120 min of aging. The fluorescence decay curve was measured with an excitation of 365 nm. The decay curve can be best fitted to a two exponential kinetic programs. The average lifetime (*τ*_av_) of as-synthesized TGA@CdS QDs is calculated to be 36.57 ns depending on the lifetimes are *τ*_1_ = 3.15, *τ*_2_ = 115.73 ns. The longer lifetime is generally assigned to the involvement of surface defects in the carrier recombination process. The shorter lifetime may be assigned to radiative depopulation due to band-edge recombination or to electro–hole recombination at the surface^38–40^.

### Selectivity and sensitivity of Co^2+^ by TGA@CdS QDs

**Figure 7:**
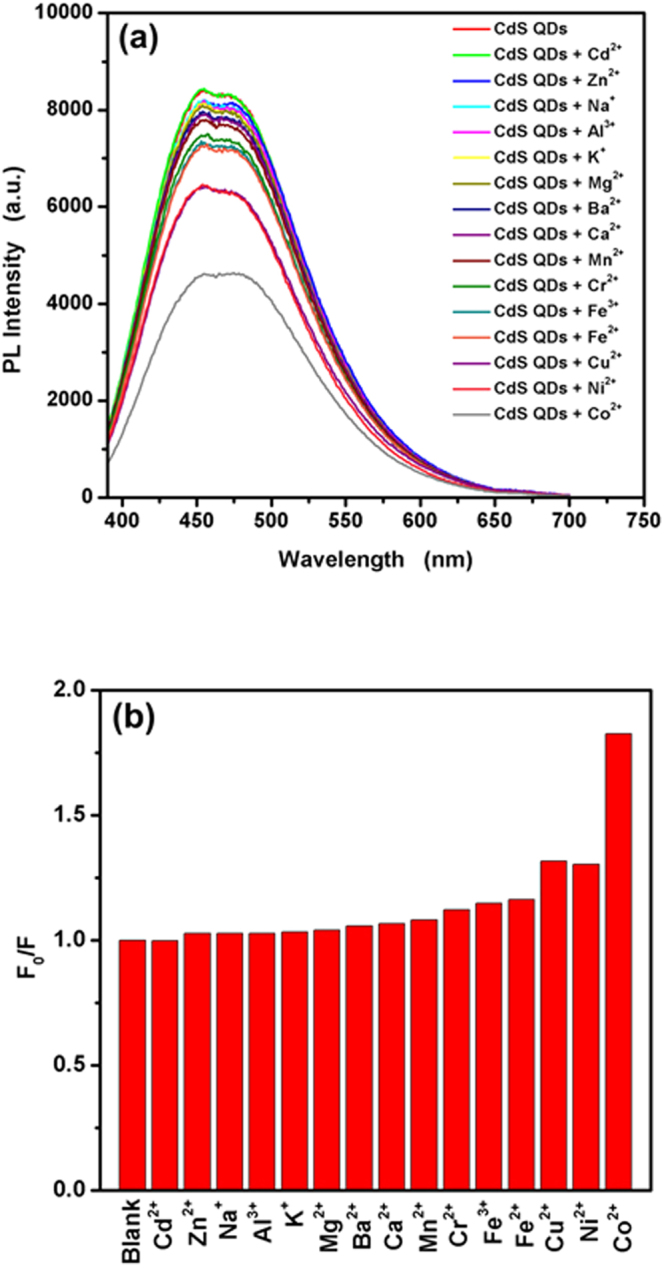
(**a**) Fluorescence spectra of TGA@CdS QDs in the presence of various cations, [M^n+^] = 50 μM, (**b**) Comparison of degree of fluorescence quenching (*F*_0_/*F*) in the presence of each cation.

To evaluate the selectivity and sensitivity of the present sensing system, 50 μM of various metal ions, including Cd^2+^, Zn^2+^, Na^+^, Al^3+^, K^+^, Mg^2+^, Ba^2+^, Ca^2+^, Mn^2+^, Cr^2+^, Fe^2+^, Fe^3+^, Cu^2+^, Ni^2+^ and Co^2+^ were added into the CdS QDs solution, and the PL responses were recorded, respectively. The PL spectra of TGA@CdS QDs before and after adding metal cation and the comparison of fluorescence intensity of TGA@CdS QDs in the absence and presence of interfering ions are shown in Fig. 7(a,b), respectively. Significant fluorescence quenching was observed with the addition of Co^2+^, while the other metal ions showed only a negligible quenching effect. Cu^2+^ and Ni^2+^ also have effect on the fluorescence, but the effect is relatively little compare with Co^2+^. The synthesized TGA@CdS QDs can be used for metal ions sensing according to metal–ligand interaction between the carboxyl acid group of TGA and metal ions^21^, and it has been observed that the CdS QDs have great sensitivity to Co^2+^.

### Fluorescence determination of Co^2+^ by TGA@CdS QDs

**Figure 8:**
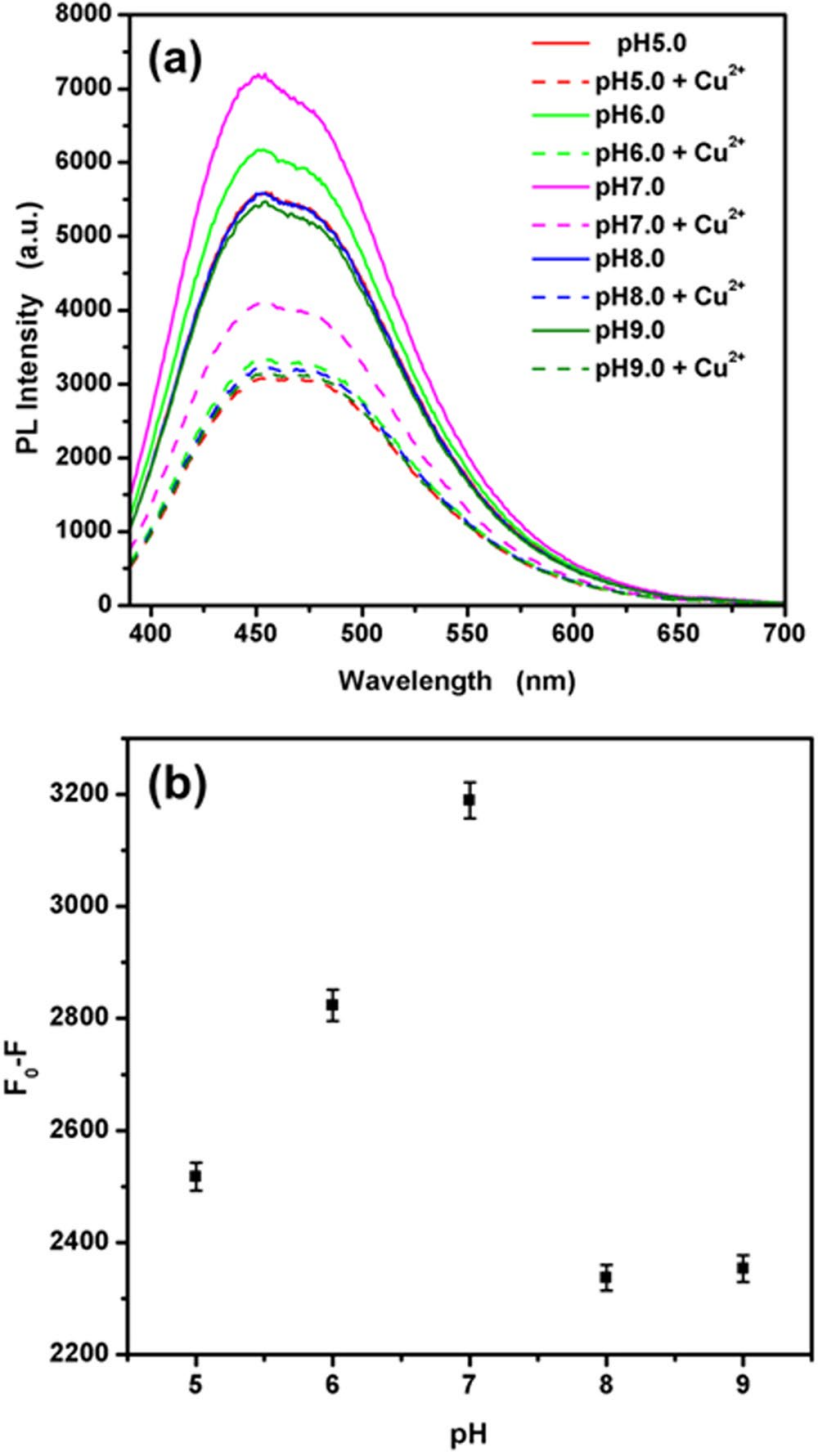
(**a**) Effect of pH on the fluorescence intensity of TGA@CdS QDs in the absence and the presence of 50 μM Co^2+^. (**b**) The fluorescence quenching in the presence of 50 μM Co^2+^.

The fluorescence intensity of TGA@CdS QDs decreased greatly when Co^2+^ was added to the QDs solution. However, the pH value of the system plays an important role, because the deprotonation process could be influenced by changing the pH value. Thus, the effect of the system pH value was studied in the range of 5.0–9.0. The pH values were adjusted by 0.3 M Tris-Hcl buffer. The results are shown in Fig. 8. The fluorescence quenching was observed at all pH value as shown in Fig. 8(a). As shown in Fig. 8(b), the quenched fluorescence intensity increases a little as the pH value varies from 5.0 to 7.0, the quenched fluorescence intensity decreasing sharply from 7.0–9.0. The reasons for it may be partial Co^2+^ coordination with free hydroxyl rather than the carboxyl group on the surface of the QDs. The maximum fluorescence quenching was obtained at pH 7.0. Therefore, a Tris-Hcl buffer of pH 7.0 was used in this work.

**Figure 9:**
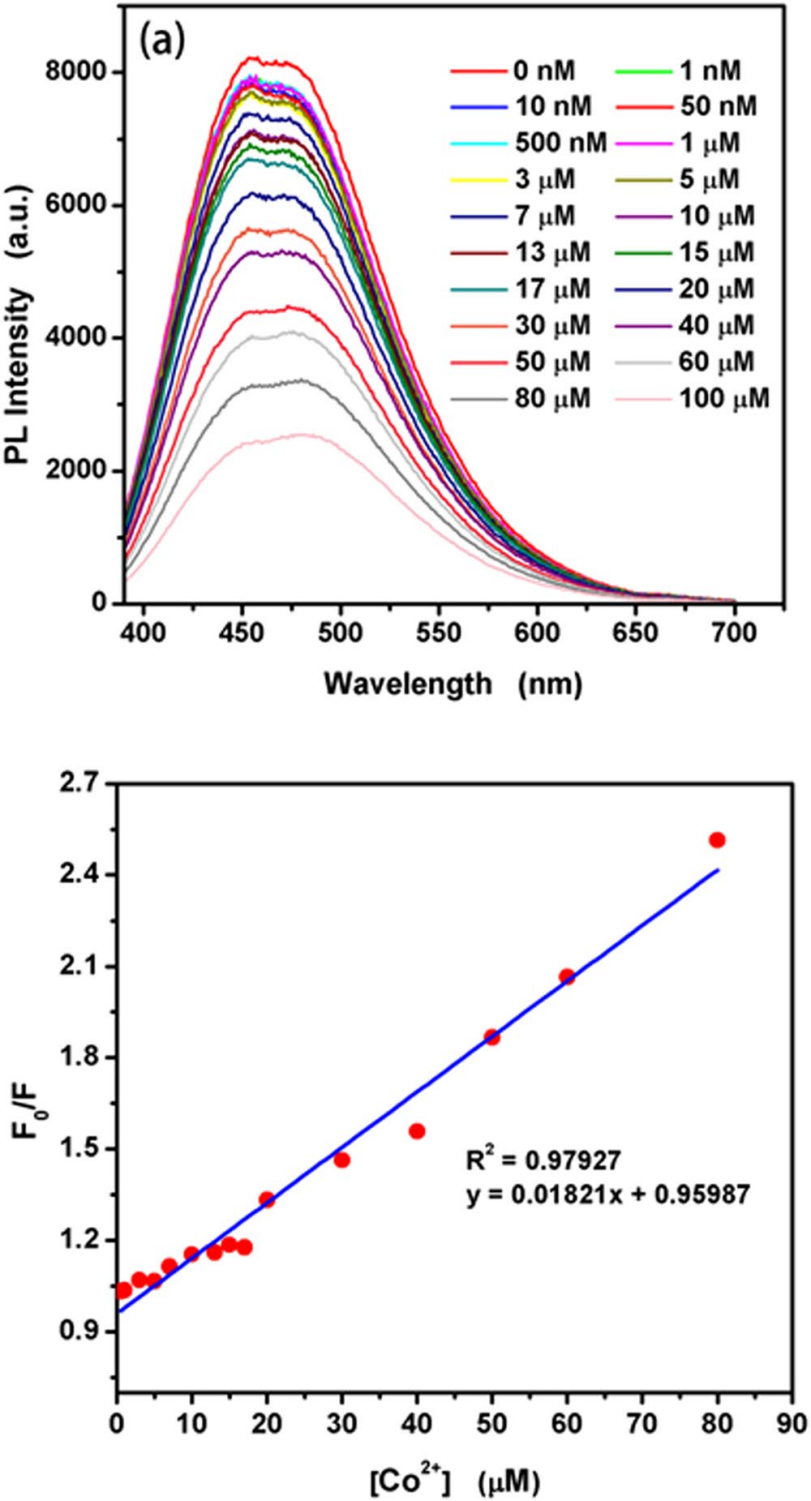
(**a**) Fluorescence spectra of as-synthesized CdS QDs solution upon addition of various concentrations of Co^2+^. (**b**) Relative emission intensity of CdS QDs in the presence of various concentrations of Co^2+^.

In order to study TGA@CdS QDs for the quantitative analysis of Co^2+^, the analytical parameters were then evaluated. The fluorescence spectra of TGA@CdS QDs in the presence of different concentrations of Co^2+^ were recorded and the results are shown in Fig. 9. Figure 9(a) shows the emission peaks remain essentially unchanged and the PL intensity gradually decreased with the increasing the concentration of Co^2+^. Figure 9(b) shows a great linear correlation (*R*^2^ = 0.97927) between the PL intensity and the concentration of Co^2+^ in the range of 0.5 to 80 μM. The limit of detection (LOD) is estimated to be 0.05 μM, based on a signal-to-noise ratio (*S/N*) of 3. The results suggested that the as-synthesized TGA@CdS QDs can be used to fluorescence detection of Co^2+^ with high sensitivity and simplicity.

### Study of the quenching mechanisms of Co^2+^ on PL emission of TGA@CdSQDs

**Figure 10:**
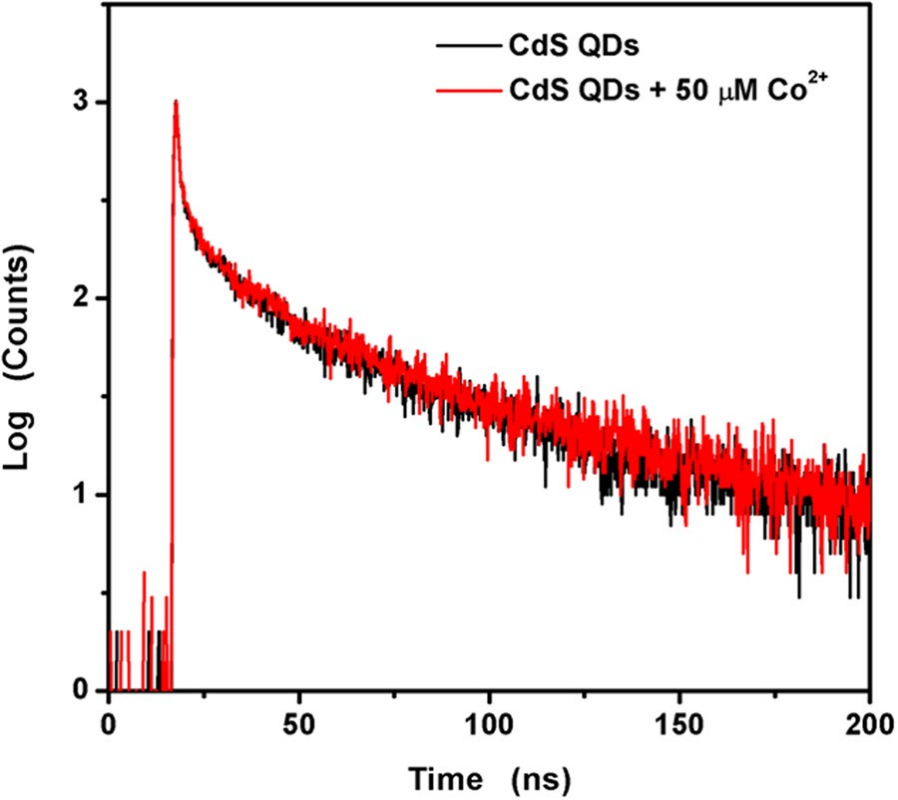
Photoluminescence lifetime decays of pure as-synthesized CdS QDs (black line) and presence of a concentration of Co^2+^ (red line).

**Figure 11:**
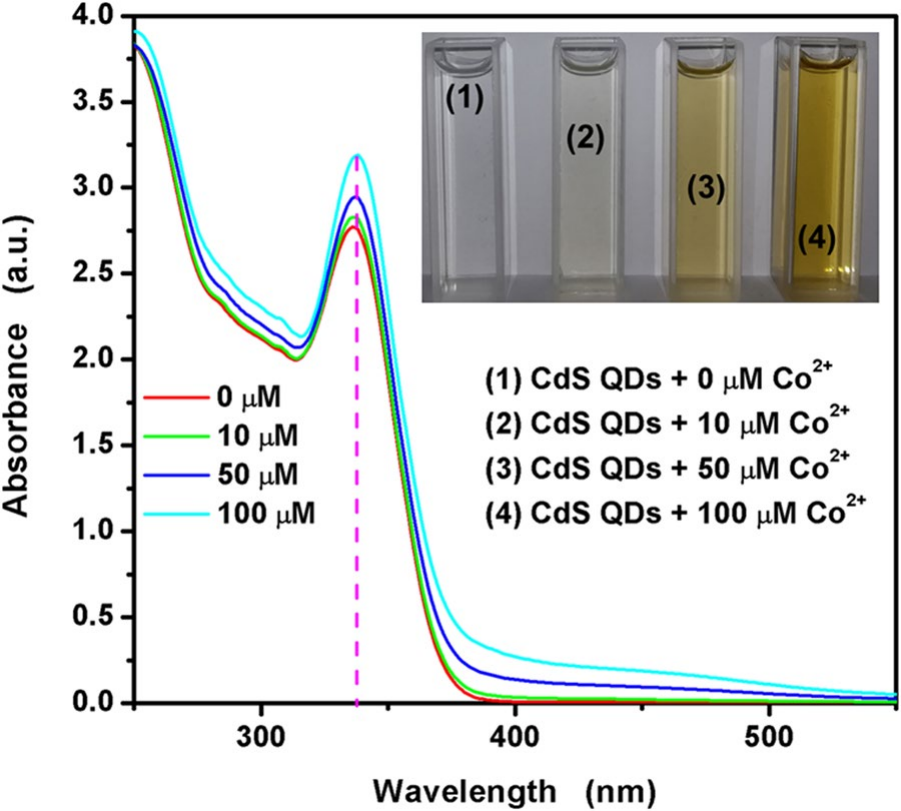
UV-vis spectra of Co^2+^ solution and as-synthesized CdS QDs with different Co^2+^ concentration.

The as-synthesized TGA@CdS QDs can be used for metal sensing based on metal–ligand interaction between the carboxyl acid group of TGA and metal ions, and it has been observed that these QDs have outstanding sensitivity towards Co^2+^^25^. In this work, the fluorescence decay curves (Fig. 10) and the UV-vis spectra (Fig. 11) of the as-synthesized CdS QDs in the absence and presence of Co^2+^ provide foundation for further study. As shown in Fig. 10, the fluorescence decay curves are does not change upon addition of Co^2+^. The fluorescence lifetime keeps constant indicates that the excitation and electron transition dynamics in CdS QDs are cannot affected by Co^2+^. Hence, there can exclude the possibility of the excited state of PL quenching and the electron transfer processes due to the bigger *K*_sp_ value of CoS than that of CdS (the p*K*_sp_ values of CoS and CdS are 20.4 and 26.1, respectively). The coordination interaction between QDs and metal ions through the carboxyl group of TGA may form some non-radiative channels for energy dissipation resulting in weakened fluorescence of QDs upon addition of Co^2+^^25,37,41^. The UV-vis spectra of the as-synthesized TGA@CdS QDs reveal the same track in the presence of Co^2+^. But a gradual increase in the absorbance of TGA@CdS QDs occurs at 325 nm with a slight red shift about 10 μM in absorbance maxima. Besides, a visual color change from colorless to yellow was observed in Fig. 11 inset. Thus the coordination interaction between QDs and Co^2+^ through the carboxyl group of TGA may result in the change of QDs with different sizes.

**Figure 12:**
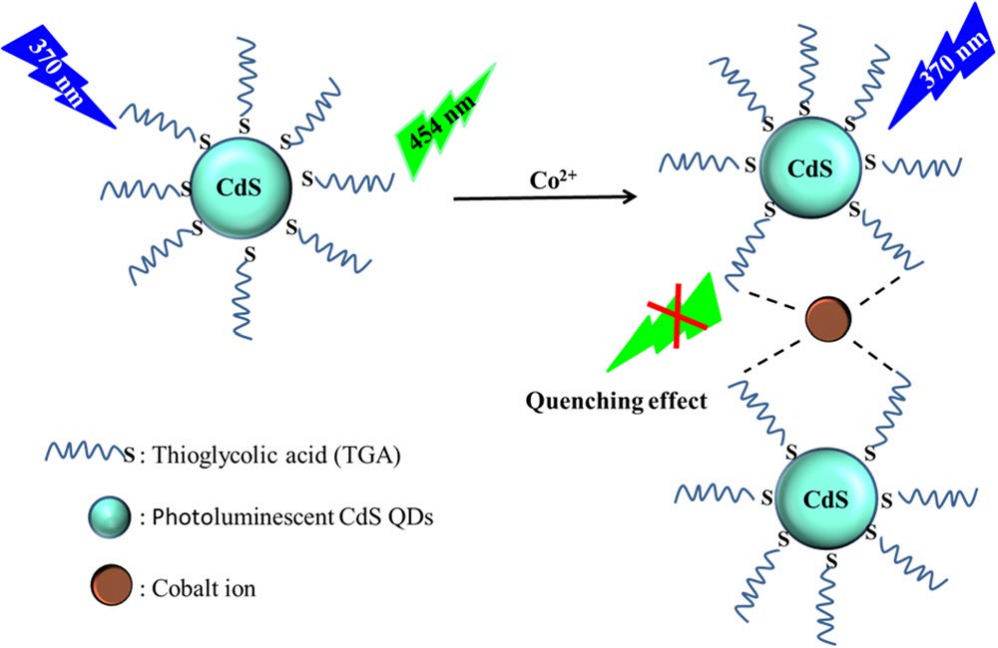
Schematic illustration of the process of Co^2+^-induced luminescence quenching of TGA@CdS QDs due to the coordination interaction between Co^2+^ and QDs through the carboxyl group of TGA.

The basic principle of as-synthesized QDs based Co^2+^ sensing system was shown in Fig. 12. In the presence of Co^2+^, the thiol groups of TGA attached to the surface of the QDs through Cd-S bonding and the negatively charged carboxyl group is easily coordination with Co^2+^. This means that the decrease of PL intensity was caused Co^2+^ could coordinate with two or more TGA@CdS QDs with a conspicuous change in absorbance and color of TGA@CdS QDs.

### Comparison the analytical merits to other Co^2+^ determination based QDs

Fluorescence determination based quantum dots for selective detection of Co^2+^ is an attractive research topic because their high sensitivity and selectivity. Several types of core quantum dots were synthesized with different surface-modified and used to as the Co^2+^ determination as summarized in Table 1. The results show that the present assay system exhibits superior selectivity and sensitivity to Co^2+^. Although the mode of detection is not different with other related determinations based fluorescence QDs, its detection limit (0.05 μM) is low which provide high sensitivity of the proposed sensor.

**Table 1:** Comparison the analytical merits of the Co^2+^ determination based fluorescence quantum dots.

Type of QDs	Surface-modified	Mode of detection	Linear range (μM)	Detection limit (μM)	Ref.
CdS QDs	N-thiophene-2-carboxamide	Fluorescence quenching	10–150	0.83	^20^
CdS QDs	L-Cysteine	Fluorescence quenching	4–80	1.13	^22^
CdS QDs	3-Mercaptopropionic acid (MPA)	Fluorescence quenching	0.01–1/1–50	0.01	^25^
CdSe QDs	L-Cysteine	Fluorescence quenching	0–20	0.1	^21^
ZnO QDs	β-Cyclodextrin	Fluorescence quenching	1–10	0.34	^19^
CdS QDs	Thioglycolic acid	Fluorescence quenching	0.5–80	0.05	This work

## Conclusions

In summary, TGA capped CdS QDs have been synthesized via a simple hydrothermal method. The coordination interaction between QDs and Co^2+^ through the carboxyl group of TGA suggested that the as-synthesized CdS QDs could be used for detecting Co^2+^. After adding Co^2+^ into the solution of QDs, the change of fluorescence spectrum can be observed. At high concentration level (>10 μM), the Co^2+^ induced color changing also can be observed. The fluorescence intensity selectively quenched by Co^2+^ due to the coordination interaction between QDs and Co^2+^ through the carboxyl group of TGA. Mechanisms of fluorescence quenching are discussed in detail. The calibration plot was linear in the range between 0.5 and 80 μM. The detection limit of the present sensor is 0.05 μM, which is quite low as desired. Besides, the Co^2+^-induced color changing of the as-synthesized QDs is particularly outstanding. The TGA@CdS QDs could provide a low cost, simple, rapid and sensitive sensing strategy for detecting trace Co^2+^.

## References

[1] Li J (2017). A tricorn-rhodamine fluorescent chemosensor for detection of Co^2+^ ions. Luminescence.

[2] Chen HJ (2016). Nitrogen doped graphene quantum dots based single-luminophor generated dual-potential electrochemiluminescence system for ratiometric sensing of Co^2+^ ion. Electrochim. Acta.

[3] Tvermoes BE (2013). Cobalt whole blood concentrations in healthy adult male volunteers following two-weeks of ingesting a cobalt supplement. Food Chem. Toxicol..

[4] Park GJ (2016). A dual chemosensor for Zn^2+^ and Co^2+^ in aqueous media and living cells: Experimental and theoretical studies. Sensor. Actuat. B.

[5] Sharma Y (2017). Lithium recycling behavior of nano-phase-CuCo_2_O_4_ as anode for lithium-ion batteries. J. Power Sources.

[6] Jia BR (2016). Synthesis of mesoporous single crystal Co(OH)_2_ nanoplate and its topotactic conversion to dual-pore mesoporous single crystal Co_3_O_4_. ACS Appl. Mater. Inter..

[7] Shi N (2015). Facile synthesis of monodisperse Co_3_O_4_ quantum dots with efficient oxygen evolution activity. Chem. Commun..

[8] Emission standard of pollutants for copper, nickel, cobalt industry. China 2010.

[9] Mohandoss S (2017). A new fluorescent PET sensor probe for Co^2+^ ion detection: computational, logic device and living cell imaging applications. RSC Adv..

[10] Ghaedi M (2008). Cloud point extraction for the determination of copper, nickel and cobalt ions in environmental samples by flame atomic absorption spectrometry. J. Hazard. Mater..

[11] Beikzadeh E (2017). Determination of trace levels of cobalt ion in different real samples using dispersive liquid–liquid microextraction followed by flame atomic absorption spectrometry. J. Food Meas. Charact..

[12] Felipe-Sotelo M (2004). Microwave-assisted extraction and ultrasonic slurry sampling procedures for cobalt determination in geological samples by electrothermal atomic absorption spectroscopy. Talanta.

[13] Wang L (2016). Simultaneous determination of copper, cobalt, and mercury ions in water samples by solid-phase extraction using carbon nanotube sponges as adsorbent after chelating with sodium diethyldithiocarbamate prior to high performance liquid chromatography. Anal. Bioanal. Chem..

[14] Bakkaus E (2006). Anion exchange liquid chromatography–inductively coupled plasma-mass spectrometry detection of the Co^2+^, Cu^2+^, Fe^3+^ and Ni^2+^ complexes of mugineic and deoxymugineic acid. J. Chromatogr. A.

[15] Hutton EA (2004). Validation of bismuth film electrode for determination of cobalt and cadmium in soil extracts using ICP–MS. Talanta.

[16] Zhao LL (2012). Determination of cadmium(II), cobalt(II), nickel(II), lead(II), zinc(II), and copper(II) in water samples using dual-cloud point extraction and inductively coupled plasma emission spectrometry. J. Hazard. Mater..

[17] Korolczuk M (2007). Determination of traces of cobalt in zinc matrix by catalytic adsorptive stripping voltammetry at bismuth film electrode. Electroanal..

[18] Morfobos, M. *et al*. Simultaneous determination of nickel(II) and cobalt(II) by square wave adsorptive stripping voltammetry on a rotating-disc bismuth-film electrode. *Anal. Chim. Acta* **519**, 57–64 (2004).

[19] Geng S (2017). Determination of cobalt(II) using β-cyclodextrin-capped ZnO quantum dots as a fluorescent probe. Microchim. Acta.

[20] Faridbod F (2015). A Novel Cobalt-Sensitive Fluorescent Chemosensor Based on Ligand Capped CdS Quantum Dots. J. Fluoresc..

[21] Ben Brahima N (2017). Interaction of L-cysteine functionalized CdSe quantum dots with metallic cations and selective binding of cobalt in water probed by fluorescence. Sensor. Actuat. B.

[22] Tedsana W (2015). A circular dichroism sensor for Ni^2+^ and Co^2+^ based on L-cysteine capped cadmium sulfide quantum dots. Anal. Chim. Acta.

[23] Zi LL (2014). Thioglycolic acid-capped CuInS_2_/ZnS quantum dots as fluorescent probe for cobalt ion detection. J. Lumin..

[24] Bian W (2014). A novel phosphorescence sensor for Co^2+^ ion based on Mn-doped ZnS quantum dots. Luminescence.

[25] Mahapatra N (2014). A single source-precursor route for the one-pot synthesis of highly luminescent CdS quantum dots as ultra-sensitive and selective photoluminescence sensor for Co^2+^ and Ni^2+^ ions. J. Mater. Chem. C.

[26] Butwong N (2013). Detection of silver(I) ion based on mixed surfactant-adsorbed CdS quantum dots. Microchim. Acta.

[27] Zhang KX (2013). Facile synthesis L-cysteine capped CdS:Eu quantum dots and their Hg^2+^ sensitive properties. Appl. Surf. Sci..

[28] Boonmee C (2016). Cysteamine capped CdS quantum dots as a fluorescence sensor for the determination of copper ion exploiting fluorescence enhancement and long-wave spectral shifts. Spectrochim. Acta A.

[29] Ganiga M (2016). An ascorbic acid sensor based on cadmium sulphide quantum dots. Anal. Bioanal. Chem..

[30] Kumar DS (2016). Optical properties of colloidal aqueous synthesized 3 Mercaptopropionic Acid stabilized CdS quantum dots. AIP. Conf. Proc..

[31] Hosseini SS (2016). Facile microwave approach for synthesis of CdS quantum dots as barrier layer for increasing dye-sensitized solar cells efficiency. J. Mater. Sci-Mater. El..

[32] Kaur G (2014). Size tuning of MAA capped CdSe and CdSe/CdS quantum dots and their stability in different pH environments. Mater. Chem. Phys..

[33] Samadi-maybodi A (2014). Aqueous synthesis and characterization of CdS quantum dots capped with some amino acids and investigations of their photocatalytic activities. Colloid. Surface. A.

[34] Brus LE (1984). Electron-electron and electron-hole interactions in small semiconductor crystallites: The size dependence of the lowest excited electronic state. J. Chem. Phys..

[35] Brus LE (1986). Electronic wave functions in semiconductor clusters: experiment and theory. J. Phys. Chem..

[36] Aboulaich A (2012). One-Pot noninjection route to CdS quantum dots via hydrothermal synthesis. ACS Appl. Mater. Inter..

[37] Wu P (2010). Ni^2+^-modulated homocysteine-capped CdTe quantum dots as a turn-on photoluminescent sensor for detecting histidine in biological fluids. Biosens. Bioelectron..

[38] Sanz M (2008). Femtosecond dynamics of CdTe quantum dots in water. J. Photochem. Photobio. A..

[39] Jagadeeswari S (2011). Photoinduced interaction between MPA capped CdTe QDs and certain anthraquinone dyes. J. Lumin..

[40] Uchihara T (2006). Subpicosecond time-resolved photoluminescence of thioglycerol-capped CdS nanoparticles in water. J. Photoch. Photobio. A.

[41] Gan ZX (2016). Photoluminescence of MoS2 quantum dots quenched by hydrogen peroxide: A fluorescent sensor for hydrogen peroxide. J. Appl. Phys..

